# Effects of Monoterpene-Based Biostimulants on Chickpea (*Cicer arietinum* L.) Plants: Functional and Molecular Insights

**DOI:** 10.3390/biology14060657

**Published:** 2025-06-05

**Authors:** Lamyae Et-Tazy, Riccardo Fedeli, Oussama Khibech, Abdeslam Lamiri, Allal Challioui, Stefano Loppi

**Affiliations:** 1 Laboratory of Applied Chemistry and Environment, Faculty of Sciences and Techniques, Hassan First University, Settat 26002, Morocco; l.et-tazy@uhp.ac.ma (L.E.-T.); abdeslam.lamiri@uhp.ac.ma (A.L.); 2 BioAgry Lab, Department of Life Sciences, University of Siena, 53100 Siena, Italy; loppi@unisi.it; 3 Laboratory of Applied and Environmental Chemistry, Faculty of Sciences, Mohammed First University, Oujda 60050, Morocco; oussama.khibech.d24@ump.ac.ma (O.K.); a.challioui@ump.ac.ma (A.C.); 4 National Biodiversity Future Center, 90121 Palermo, Italy

**Keywords:** cineole, carvacrol, thymol, foliar application, sustainable agriculture, plant biochemical response

## Abstract

This study evaluated the physiological and biochemical responses of chickpea (*Cicer arietinum* L.) to foliar application of cineole, carvacrol, and thymol at concentrations of 500 and 1000 ppm. Carvacrol at 1000 ppm significantly enhanced fresh biomass (+15.4%) and aerial biomass (+46.2%), whereas thymol significantly reduced plant height (−20.2%) and overall biomass, yet notably increased chlorophyll content (+23.3%) and vitamin C levels (+41.4%) at the same concentration. Cineole significantly improved antioxidant capacity by increasing total phenolic content (+15.5% at 1000 ppm) and total flavonoid content (+19.1% at 500 ppm), but simultaneously decreased soluble protein synthesis and chlorophyll content (−39% at 500 ppm). Mineral analysis showed notable increases in calcium content following treatment with cineole (+30.5% at 1000 ppm) and carvacrol (+32% at 500 ppm), while thymol at 1000 ppm significantly reduced phosphorus, potassium, manganese, iron, copper, and zinc accumulation. Molecular docking and dynamic simulations revealed strong interactions of thymol and carvacrol with essential enzymes, specifically ascorbate peroxidase and phenylalanine ammonia-lyase, which are involved in antioxidant and phenolic metabolism pathways. These molecular interactions suggest potential contributions of thymol and carvacrol to plant stress resilience mechanisms, although further experimental validation is needed to confirm their roles in vivo. These findings emphasize the importance of optimizing monoterpene concentrations, indicating that carefully calibrated treatments could effectively enhance chickpea growth, nutritional quality, and stress tolerance within sustainable agricultural practices.

## Simple Summary

This study evaluated the effects of foliar application of three monoterpenes—cineole, carvacrol, and thymol—at concentrations of 500 and 1000 ppm on the growth and physiological responses of chickpea plants. Carvacrol significantly increased fresh and shoot biomass, whereas thymol reduced plant height and overall biomass but enhanced chlorophyll and vitamin C content. Cineole enhanced the antioxidant capacity of chickpea plants by increasing phenolic and flavonoid content but negatively affected soluble protein and chlorophyll synthesis at certain concentrations. Mineral analyses indicated increased calcium accumulation in response to cineole and carvacrol treatments, while thymol reduced the accumulation of essential nutrients, including phosphorus, potassium, and several micronutrients. Molecular docking and dynamic simulation analyses demonstrated strong binding interactions between thymol and carvacrol with key enzymes, notably ascorbate peroxidase and phenylalanine ammonia-lyase, suggesting their possible roles in antioxidant defense and phenolic metabolism pathways. These results highlight that carefully optimized applications of monoterpenes have potential to enhance chickpea growth, nutritional quality, and stress resilience within sustainable agricultural practices.

## 1. Introduction

Chickpea (*Cicer arietinum* L.) is a globally significant grain legume cultivated predominantly in semi-arid and arid regions due to its nutritional importance and adaptability to marginal environments [1,2]. It represents an essential dietary component, particularly in developing countries, because of its high protein, dietary fiber, vitamin, and mineral content [3]. Despite its agricultural relevance, chickpea productivity is frequently constrained by abiotic stresses, including drought, heat, and salinity, negatively influencing growth, yield, and nutritional quality [4]. In countries such as Morocco and Italy, chickpea cultivation is widespread yet significantly limited by these stresses, resulting in persistently low yields and considerable dependence on imports to satisfy domestic demand [5,6,7,8]. Consequently, there is an urgent need to develop sustainable agronomic strategies to mitigate these stress factors, particularly drought and heat stresses predominant in Morocco [5], and combined drought and salinity stresses commonly affecting chickpea production in Italy [9].

Recently, essential oils (EOs) and their bioactive components have emerged as promising agents for enhancing plant resilience under adverse conditions [10,11]. EOs, complex mixtures of volatile compounds extracted from aromatic plants, possess strong antimicrobial, antioxidant, and stress-mitigating properties [12,13,14]. Monoterpenes, active constituents of EOs, have attracted attention as natural biostimulants capable of improving plant growth and stress tolerance through various biochemical and physiological mechanisms [15]. These compounds modulate plant hormone levels, antioxidant defense systems, and metabolic pathways involved in stress responses, nutrient uptake, and photosynthesis [16,17]. Studies indicate that monoterpenes stimulate the synthesis of secondary metabolites, enhance chlorophyll content, and activate antioxidant enzymes, thereby alleviating oxidative damage under stressful conditions [18,19,20]. Furthermore, their small molecular size and lipophilic nature facilitate effective penetration and rapid absorption into plant tissues, emphasizing their potential effectiveness in foliar applications [21]. Therefore, exploring monoterpenes as biostimulants represents a sustainable strategy for improving chickpea growth, productivity, and nutritional quality [22].

Among monoterpenes, cineole, carvacrol, and thymol exhibit particularly notable bioactivities. Cineole, a major constituent of eucalyptus (*Eucalyptus globulus* Labill.) and rosemary (*Salvia rosmarinus* Spenn. L.) oils, demonstrates strong antifungal and allelopathic activities, enhancing plant defense mechanisms [23,24]. Carvacrol, predominantly found in oregano (*Origanum vulgare* L.) and thyme (*Thymus vulgaris* L.) oils, exhibits antifungal properties and promotes plant growth, improving vigor and stress tolerance [25,26]. Thymol, another major component of thyme oil, has been shown to enhance stress tolerance, antioxidant capacity, and growth-related metabolic processes [27,28].

Although the bioactivities of these monoterpenes are recognized, direct foliar applications on legumes, particularly chickpea, remain largely unexplored. Current research predominantly focuses on seed treatments or in vitro assays, providing limited insight into the effects of monoterpenes on plant foliage [29,30]. To date, no comprehensive study has systematically evaluated the physiological, biochemical, and molecular effects of monoterpene foliar treatments specifically on chickpea plants. Additionally, the precise molecular interactions between these bioactive compounds and plant metabolic pathways remain unclear. Integrating computational approaches, such as molecular docking and molecular dynamics simulations, with empirical plant assays can effectively elucidate these molecular interactions [31].

Transcriptomic analyses complement these approaches by identifying gene-regulatory effects of monoterpenes associated with hormone signaling and metabolic adjustments [32]. Proteomic studies have demonstrated shifts in protein abundance and enzyme activity involved in photosynthesis and stress adaptation, providing biochemical evidence supporting improved resilience [33]. Metabolomic investigations have further revealed alterations in primary and secondary metabolite profiles, enhancing nutritional value and stress tolerance [34]. Integrating these molecular insights can significantly advance the effective application of monoterpenes in sustainable chickpea cultivation practices.

This study aims to comprehensively evaluate the physiological, biochemical, and nutritional impacts of cineole, carvacrol, and thymol, applied foliarly at concentrations of 500 and 1000 ppm, on chickpea under controlled conditions. Additionally, molecular docking and molecular dynamics simulations are employed to investigate the interactions between these compounds and key enzymes involved in antioxidant defense, chlorophyll biosynthesis, and stress-response pathways. This integrative approach addresses existing knowledge gaps, facilitating the development of innovative and sustainable agronomic practices designed to enhance chickpea productivity and nutritional quality under abiotic stress conditions.

## 2. Materials and Methods

### 2.1. Major Compounds

**Figure 1:**
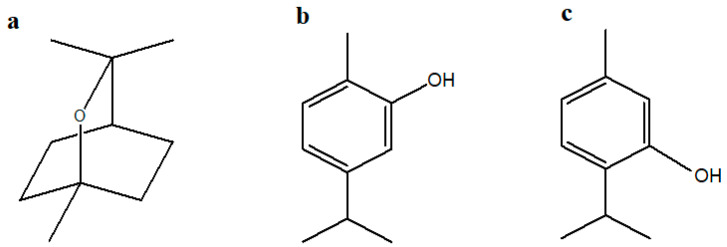
Chemical structures of monoterpenes created using ChemDraw Pro (version 8.0): (**a**) Cineole; (**b**) Carvacrol; (**c**) Thymol.

Three highly purified monoterpenes (Figure 1), specifically cineole (≥98%), carvacrol (≥97%), and thymol (≥99%), were selected to assess their efficacy as foliar treatments in enhancing chickpea growth, biochemical characteristics, and nutritional quality. All compounds were purchased from Fluka Chemica (Buchs, Switzerland).

### 2.2. Experimental Design

Chickpea (*Cicer arietinum* L.) seeds used in this study were obtained from organically cultivated plants sourced from a nursery in Crete Senesi, Tuscany, Italy. Prior to sowing, seeds were sterilized by immersion in a 5% sodium hypochlorite (NaClO) solution for 10 min under continuous agitation, followed by three thorough rinses with sterile distilled water, each lasting 5 min, to completely remove residual disinfectant [35].

The experiment was carried out under controlled conditions in a growth chamber during February 2025. Sterilized chickpea seeds (n = 42, six replicates per treatment) were individually sown at an approximate depth of 1 cm in pots filled with a standard horticultural substrate (Vigor Plant Srl, Piacenza, Italy), the chemical characterization of which was previously reported by Maresca et al. (2024) [36]. One week after germination, uniform seedlings were selected and individually transplanted into new pots. Growth chamber conditions were maintained at a temperature of 22 ± 2 °C, relative humidity of 70 ± 1%, and a consistent photoperiod consisting of 16 h of light (250 μmol m^−2^ s^−1^ PAR) followed by 8 h of darkness [37]. Plants were irrigated every two days with tap water to maintain soil moisture at approximately 70% of field capacity [38].

Three monoterpenes—cineole, carvacrol, and thymol—were prepared at two concentrations (500 ppm and 1000 ppm). These concentrations were selected based on a previous study involving foliar application of *Rosmarinus officinalis* essential oil on tomato seedlings [39]. Treatment solutions were prepared by dissolving 500 µL (500 ppm) or 1000 µL (1000 ppm) of each pure monoterpene per liter of distilled water (dH_2_O), considering the approximate density (~1 g/mL) of the monoterpenes. Tween 20 (10 µL/mL) was added as an emulsifier to ensure uniform dispersion and improve adherence of the hydrophobic monoterpenes onto leaf surfaces [40]. Solutions were thoroughly homogenized prior to application.

Foliar spraying commenced three weeks after seedling emergence and continued once weekly for four consecutive weeks until the conclusion of the experiment [41]. Foliar treatments were applied using a portable handheld sprayer to ensure uniform and complete coverage of the leaf surfaces.

### 2.3. Biometric Parameters and Chlorophyll Content

Biometric parameters were recorded immediately prior to plant harvesting. Plant height was measured from the soil surface to the apex of the shoot using a ruler [42]. Fresh biomass and aerial biomass of the total aboveground portion were determined to indicate plant growth and water status. Fresh biomass was recorded immediately upon harvest, whereas aerial biomass was determined after oven-drying the harvested plant material at 40 °C for 24 h, according to previously established protocols [43]. Leaf chlorophyll content was assessed using a portable, non-destructive chlorophyll content meter (CCM-300, Opti-Sciences Inc., Hudson, NH, USA). Measurements were conducted on the youngest fully expanded leaves, with three readings obtained per leaf for each plant. Chlorophyll content was expressed on a leaf surface area basis (mg m^−2^) [44].

### 2.4. Mineral Elements

The concentrations of mineral elements in whole chickpea plants were analyzed using an Olympus Vanta Series C portable X-ray fluorescence (XRF) analyzer (Olympus Corp., Waltham, MA, USA), equipped with an Ag-anode X-ray tube operating at excitation energies ranging from 15 to 40 kV and a large-area silicon drift detector, as described previously [45]. Approximately 1 g of dried, powdered plant material was placed into a plastic sample cup, positioned within the instrument’s measurement compartment, and analyzed using the “Soil” mode setting. Each sample underwent three sequential measurements with an acquisition time of 20 s per beam. The analyzed elements included calcium (Ca), chlorine (Cl), copper (Cu), iron (Fe), potassium (K), manganese (Mn), phosphorus (P), sulfur (S), and zinc (Zn). The minimum detection limits were as follows: Ca (30 mg/kg), Cl (30 mg/kg), Cu (3 mg/kg), Fe (4 mg/kg), K (20 mg/kg), Mn (10 mg/kg), P (30 mg/kg), S (30 mg/kg), and Zn (2 mg/kg). Analytical accuracy was validated using 14 certified plant reference matrices [46]. Results were expressed on a dry weight basis (mg/kg DW).

### 2.5. Antioxidant Compounds

The total content of antioxidant compounds in chickpea plants was determined according to the protocol described by Azarnejad et al. (2024) [47]. Chickpea plant samples were initially sectioned into small fragments, oven-dried at 40 °C for 24 h, and subsequently ground into a fine powder using an Ultra-Turrax homogenizer (IKA A10, KA-Werke GmbH & Co. KG, Staufen im Breisgau, Germany). Approximately 0.5 g of powdered sample was mixed with 5 mL of 80% methanol and homogenized for 30 min. The homogenate was then stored at 4 °C in darkness for 48 h. Following extraction, the mixture was filtered through Whatman No. 1 filter paper, producing a clear extract suitable for subsequent analyses.

#### 2.5.1. Total Polyphenol Content

Total polyphenol content was assessed using a refined colorimetric method based on the Folin–Ciocalteu assay [48]. Briefly, 125 μL of plant extract was diluted with 2 mL distilled water, followed by the addition of 125 μL Folin–Ciocalteu reagent. After mixing and allowing the reaction to proceed for 3 min, 1.25 mL of 7% sodium carbonate (Na_2_CO_3_) and an additional 1 mL of distilled water were added. The reaction mixture was incubated in darkness for 90 min, after which absorbance was measured at 760 nm using a UV-Vis spectrophotometer (Agilent 8453, Santa Clara, CA, USA). Results were quantified against a gallic acid standard curve (5–300 μg/mL) and expressed as milligrams of gallic acid equivalents per gram of dry weight (mg GAE/g DW; GAE: gallic acid equivalents; DW: dry weight).

#### 2.5.2. Total Flavonoid Content

Total flavonoid content was determined using an aluminum chloride-based colorimetric method [49]. Briefly, 10 μL of the plant extract was diluted initially with 200 μL distilled water. Subsequently, 75 μL of 5% sodium nitrite (NaNO_2_) was added, and the mixture was incubated in darkness for 5 min. Following incubation, 75 μL of 10% aluminum chloride (AlCl_3_) was added, mixed thoroughly, and incubated in darkness for another 5 min. Finally, 500 μL of 1 N sodium hydroxide (NaOH) was introduced. The resulting solution was homogenized thoroughly and incubated in darkness for an additional 15 min. Absorbance was measured at 415 nm using a UV-Vis spectrophotometer (Agilent 8453, Santa Clara, CA, USA). Flavonoid concentrations were calculated using a quercetin standard curve (12.5–150 μg/mL) and expressed as milligrams of quercetin equivalents per gram of dry weight (mg QE/g DW; QE: quercetin equivalents).

### 2.6. Total Soluble Proteins

The total soluble protein content was determined using a Bradford assay with minor modifications [50]. Briefly, approximately 50 mg of powdered chickpea plant material was homogenized in 5 mL of distilled water and centrifuged at 4000 rpm for 5 min. Subsequently, 0.2 mL of the resulting supernatant was mixed with 0.8 mL Bradford reagent (Sigma-Aldrich, St. Louis, MO, USA). Absorbance was measured at 595 nm using a UV-Vis spectrophotometer (Agilent 8453, Agilent Technologies, Inc., Santa Clara, CA, USA). Protein concentrations were calculated based on a bovine serum albumin (BSA) standard curve (20–80 μg/mL) and expressed as milligrams of BSA equivalents per gram of dry weight (mg BSA eq/g DW; BSA eq: bovine serum albumin equivalents).

### 2.7. Vitamin C (Ascorbic Acid)

Vitamin C content was quantified following the protocol described by Celletti et al. (2023) [51] with slight modifications. Briefly, 200 mg of fresh leaf material was homogenized in 0.8 mL of chilled 10% (*w*/*v*) trichloroacetic acid (TCA). The resulting homogenate was filtered through gauze, incubated in an ice bath for 5 min, and subsequently centrifuged at 3000 rpm for 5 min. Then, 0.4 mL of the supernatant was diluted with 1.6 mL distilled water, followed by the addition of 0.2 mL of 0.2 M Folin–Ciocalteu reagent (Carlo Erba, Cornaredo, Milan, Italy). The mixture was incubated in darkness for 10 min, after which absorbance was measured at 760 nm using a UV–Vis spectrophotometer (Agilent 8453, Santa Clara, CA, USA). Vitamin C concentration was calculated using a calibration curve prepared from known concentrations (5–20 µg) of L-ascorbic acid (BioXtra, ≥99.0%, crystalline), derived from a 100 µg·mL^−1^ stock solution.

### 2.8. PyRx-Based Molecular Docking: Preparation, Validation, and Visualization

Five target proteins (PDB codes: 6L1H, 1I89, 6AT7, 1IYN, and 3OGH) were selected based on their critical roles in chickpea physiology and abiotic stress responses. Specifically, Protochlorophyllide Reductase (6L1H) participates in chlorophyll biosynthesis, influencing photosynthetic efficiency and plant growth [52]; Chalcone Synthase (1I89) initiates flavonoid biosynthesis, crucial for antioxidant defense mechanisms [53]; Phenylalanine Ammonia-Lyase (6AT7) catalyzes a key step in phenylpropanoid metabolism, essential for synthesizing defensive phenolic compounds [54]; Ascorbate Peroxidase (1IYN) acts as a primary enzyme managing oxidative stress by scavenging reactive oxygen species [55]; and Ferritin (3OGH) regulates iron storage, contributing to stress tolerance and metal homeostasis [56].

All molecular docking computations were conducted using PyMOL [57] (version 3.1), AutoDock Tools [58] (ADT, MGLTools version 1.5.7), and PyRx [59] (version 0.9.8). Initially, the three ligands of interest were obtained in SMILES format and converted into three-dimensional SDF files. These files were then imported into PyRx, where each ligand underwent energy minimization using default parameters. Protein preparation involved retrieving crystal structures of the five target proteins from the Protein Data Bank (PDB codes: 6L1H, 1I89, 6AT7, 1IYN, and 3OGH). Each protein structure was visually inspected in PyMOL, and non-essential molecules (e.g., water or cofactors not involved in active sites) were removed as needed. Subsequently, proteins were loaded into AutoDock Tools, where polar hydrogens were added, Kollman charges assigned, and structures saved in PDBQT format. Ligand files were similarly converted into PDBQT format within PyRx following energy minimization.

Docking validation was performed for two proteins, 6L1H and 1IYN, by theoretical re-docking procedures (reproducing known ligand binding poses or original ligand positions), yielding RMSD values of 1.747 Å and 0.171 Å, respectively—both below the widely accepted 2.0 Å threshold indicative of successful pose prediction. Docking grid parameters were set in PyRx with an exhaustiveness value of 8 for validated targets. Specifically, the grid box for 6L1H was centered at (x = 37.79, y = 4.79, z = 86.00), with dimensions (x = 72.21, y = 54.76, z = 54.56); for 1IYN, the grid was centered at (x = −0.28, y = 35.18, z = 24.65), with dimensions (x = 11.44, y = 15.15, z = 7.67). All remaining docking parameters were maintained at default settings unless otherwise specified.

After docking runs, the resulting poses were evaluated by ranking the configurations according to their binding affinity scores (expressed in kcal/mol). The top-ranked ligand poses were visualized using Discovery Studio [60] (version 2021) to identify and illustrate key molecular interactions. This integrated workflow, encompassing ligand and protein preparation, theoretical docking validation, computational docking, and final visualization, ensured both the reliability of docking outcomes and a comprehensive assessment of ligand–protein interactions at the molecular level.

### 2.9. Implementation of Molecular Dynamics Simulations Using GROMACS

The protein structure file (P.pdb) was initially prepared using Chimera to remove any redundant molecules and correct potential structural inconsistencies. The processed structure was subsequently prepared with the GROMACS “gmx pdb2gmx” tool under the CHARMM27 force field. This step generated the required topology files, added missing hydrogens, and assigned appropriate protonation states. For ligand preparation, each ligand was individually parameterized using the SwissParam online platform, generating topology (.itp) and coordinate (.pdb) files. The resulting ligand structures (LIG.pdb) from SwissParam were then converted into GROMACS-compatible coordinate format (LIG.gro). Following parameterization, each ligand was merged with the protein to form the protein–ligand complex. The resulting complex was placed into a cubic simulation box, solvated with TIP3P water, and neutralized by adding ions to balance the overall charge. Energy minimization and equilibration were conducted using GROMACS (version 2021.4) following established protocols and default parameters [61]. Finally, a 100 ns production molecular dynamics simulation was performed, with periodic recording of atomic coordinates and velocities. This procedure allowed assessment of the stability, structural dynamics, and critical ligand–protein interactions within each complex.

### 2.10. Statistical Analysis

Data normality was verified using the Shapiro–Wilk test (*p* > 0.05). Differences among treatments regarding plant growth parameters, mineral element concentrations, total phenolic content, total flavonoid content, and total soluble protein content were evaluated using one-way analysis of variance (ANOVA). Where significant differences were detected, Tukey’s post hoc test was conducted for multiple comparisons at a significance threshold of *p* < 0.05. Statistical analyses were performed using IBM SPSS Statistics (version 22), and graphs were prepared using OriginPro 2025.

## 3. Results

### 3.1. Plant Growth Parameters

**Figure 2:**
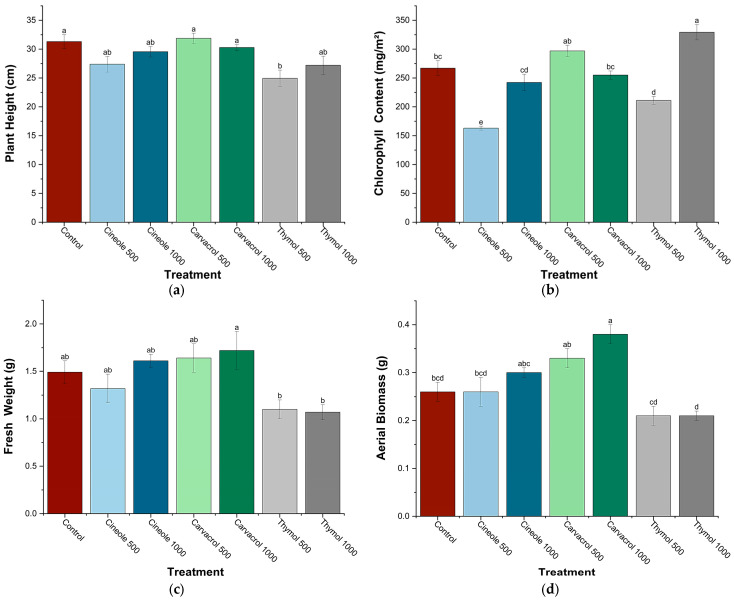
Effect of major compounds (cineole, carvacrol, and thymol) at two concentrations (500 and 1000 ppm) on plant height (**a**), chlorophyll content (**b**), fresh weight (**c**), and aerial biomass (**d**) of chickpea plants (*Cicer arietinum* L.). Values are presented as means ± SE (n = 6). Within each parameter, values followed by different letters differ significantly (*p* < 0.05) according to Tukey’s post hoc test.

Significant variations in chickpea plant growth parameters were observed following foliar treatments with cineole, carvacrol, and thymol at concentrations of 500 and 1000 ppm (Figure 2). Plant height significantly decreased in response to thymol treatment at 500 ppm (−20.2%). Chlorophyll content exhibited a significant increase following treatment with thymol at 1000 ppm (+23.3%) but significantly decreased after cineole treatment at 500 ppm (−39%). Regarding fresh weight, an indicator of plant water retention capacity and overall vigor, a significant enhancement was observed exclusively with carvacrol at 1000 ppm (+15.4%), whereas thymol treatments significantly reduced fresh weight at both concentrations tested (−26.2% at 500 ppm and −28.2% at 1000 ppm). Aerial biomass significantly increased in plants treated with carvacrol at both concentrations (+26.9% at 500 ppm and +46.2% at 1000 ppm), whereas thymol significantly reduced aerial biomass at 1000 ppm (−23.1%).

### 3.2. Mineral Element Concentrations

Significant variations in mineral element concentrations were observed in chickpea plants following foliar treatments with cineole, carvacrol, and thymol at concentrations of 500 and 1000 ppm (Table 1). Phosphorus (P) significantly decreased in response to carvacrol at 1000 ppm (−22%) and thymol at 1000 ppm (−19.7%). Sulfur (S) concentrations significantly declined across all treatments, with the most pronounced reduction observed at 1000 ppm cineole (−31%). Chlorine (Cl) content significantly increased with cineole at 1000 ppm (+33%) but decreased with thymol at 1000 ppm (−20.2%). Potassium (K) levels significantly declined under all treatments, particularly with thymol at 1000 ppm (−21.6%). Calcium (Ca) significantly increased in plants treated with cineole at 1000 ppm (+30.5%) and carvacrol at 500 ppm (+32%), but significantly decreased with carvacrol at 1000 ppm (−11.2%) and thymol at 1000 ppm (−11.7%). Manganese (Mn) content significantly increased with carvacrol at 500 ppm (+7.3%), yet significantly decreased with thymol at 1000 ppm (−49.2%). Iron (Fe) significantly decreased following thymol application at 1000 ppm (−51.2%). Copper (Cu) levels significantly decreased with thymol at 1000 ppm (−36.1%). Zinc (Zn) content significantly increased with thymol at 500 ppm (+16.2%) but significantly decreased at 1000 ppm thymol (−41.8%).

### 3.3. Phytochemical and Biochemical Parameters

**Figure 3:**
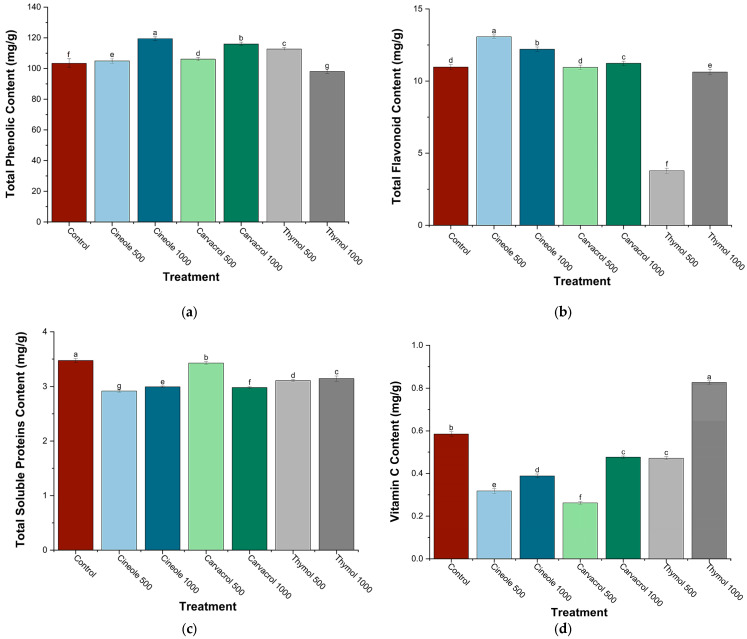
Total phenolic (**a**), flavonoid (**b**), soluble protein (**c**), and vitamin C (**d**) contents (mg g^−1^) in chickpea plants treated with major monoterpenes (cineole, carvacrol, and thymol) at two concentrations (500 and 1000 ppm). Data are presented as means ± SE (n = 6). Bars labeled with different letters indicate significant differences (*p* < 0.05) according to Tukey’s post hoc test.

The total phenolic content (TPC) exhibited significant variations in chickpea plants treated with cineole, carvacrol, and thymol at concentrations of 500 and 1000 ppm (Figure 3a). Cineole induced a concentration-dependent increase in TPC, ranging from a slight enhancement at 500 ppm (+1.4%) to a substantial elevation at 1000 ppm (+15.5%). Similarly, carvacrol treatment resulted in incremental increases, modest at 500 ppm (+2.6%) and notably higher at 1000 ppm (+12.1%). Conversely, thymol exhibited contrasting effects, with a marked increase at 500 ppm (+9%) but a notable decrease at 1000 ppm (−5.2%), presenting the lowest TPC among all treatments. Overall, cineole at 1000 ppm was most effective in enhancing TPC, whereas thymol at the same concentration decreased TPC compared to control plants.

Significant differences in total flavonoid content (TFC) were observed among treatments with cineole, carvacrol, and thymol at 500 and 1000 ppm (Figure 3b). Cineole significantly increased TFC at both concentrations tested, with the highest enhancement at 500 ppm (+19.1%) and a slightly lower but significant increase at 1000 ppm (+11.3%). Carvacrol at 1000 ppm showed a modest yet significant increase in TFC (+2.4%). In contrast, thymol significantly reduced TFC at both concentrations, with a pronounced decrease at 500 ppm (−65.5%) and a smaller yet significant reduction at 1000 ppm (−3.2%).

Total soluble protein content showed significant variations following treatment with cineole, carvacrol, and thymol at concentrations of 500 and 1000 ppm (Figure 3c). The highest protein content was recorded in control plants, while all treatments caused significant reductions. Among the treatments, carvacrol at 500 ppm maintained protein levels closest to control (−1.4%), whereas at 1000 ppm, carvacrol caused a larger significant decrease (−14.2%). Thymol treatments significantly reduced protein content at both concentrations (−10.5% at 500 ppm and −9.2% at 1000 ppm). Cineole induced the most pronounced reductions, significantly decreasing protein content by −16.1% at 500 ppm and −13.8% at 1000 ppm.

Vitamin C content varied significantly in response to cineole, carvacrol, and thymol treatments at concentrations of 500 and 1000 ppm (Figure 3d). Thymol at 1000 ppm significantly increased vitamin C content (+41.4%), achieving the highest level among all treatments. Conversely, cineole significantly reduced vitamin C content at both concentrations tested, with a more pronounced reduction at 500 ppm (−45.7%) than at 1000 ppm (−33.5%). Carvacrol at 500 ppm caused the most substantial decrease (−55.1%), resulting in the lowest vitamin C content measured, while a moderate yet significant reduction was observed at 1000 ppm (−18.5%). Additionally, thymol at 500 ppm significantly lowered vitamin C content (−19.3%), although to a lesser extent compared to cineole and carvacrol at their respective lower concentrations.

### 3.4. Docking of Monoterpenes with Chickpea Enzymes

**Figure 4:**
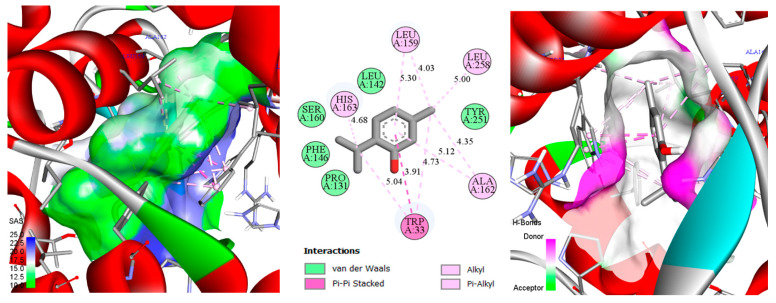
2D/3D 2D/3D depiction of the thymol–ascorbate peroxidase (1IYN) complex, highlighting the solvent-accessible surface (SAS) and key molecular interactions.

**Figure 5:**
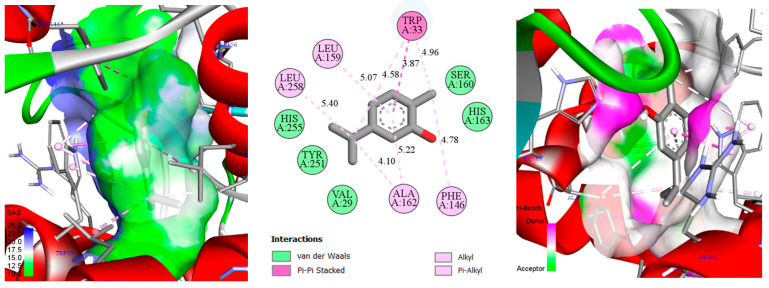
Two-dimensional/three-dimensional representation of the carvacrol–ascorbate peroxidase (1IYN) complex, showcasing the solvent-accessible surface (SAS) and key molecular interactions.

**Figure 6:**
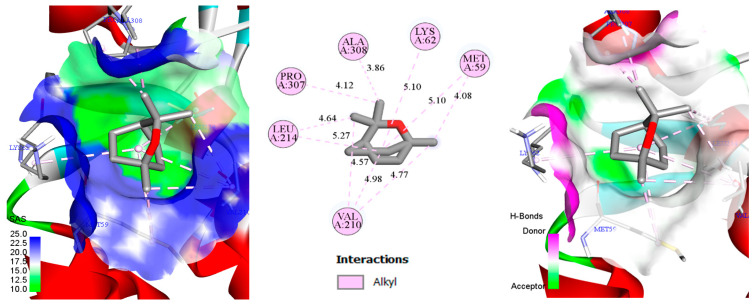
Two-dimensional/three-dimensional depiction of the cineole–protochlorophyllide reductase (6L1H) complex, highlighting the solvent-accessible surface (SAS) and key molecular interactions.

Docking scores (kcal/mol) for thymol, carvacrol, and cineole against the five selected enzyme targets are presented in Table 2. These docking values reflect the predicted binding affinity between each ligand (thymol, carvacrol, and cineole) and the respective target proteins, with more negative scores generally indicating stronger interactions. Thymol exhibited notably strong binding affinity towards enzymes 6AT7 and 1IYN (both −6.7 kcal/mol), suggesting high affinity toward phenylalanine ammonia-lyase and ascorbate peroxidase, two enzymes crucially involved in plant defense and oxidative stress management. Carvacrol displayed similar binding trends, with docking scores of −6.5 kcal/mol for both 6AT7 and 1IYN, indicating comparable interactions with these targets. Cineole showed stronger affinity towards 6L1H (−6.1 kcal/mol) and 1IYN (−5.7 kcal/mol). Docking scores for 3OGH were more moderate (ranging from −4.7 to −5.3 kcal/mol), indicating comparatively weaker interactions. Given the experimentally validated co-crystal structure available for 1IYN, this protein was selected over 6AT7 for detailed analysis, despite similar docking scores for thymol and carvacrol. Consequently, to comprehensively illustrate binding modes (including hydrogen bonds, hydrophobic interactions, etc.) and to support computational analysis with experimental validation, the thymol–1IYN, carvacrol–1IYN, and cineole–6L1H complexes were selected for detailed 2D and 3D visualizations (Figure 4, Figure 5 and Figure 6). This integrative approach, combining quantitative docking scores, crystallographic validation, and detailed structural analysis, provides deeper insights into the potential of these monoterpenes to modulate key metabolic pathways in chickpea plants.

## 4. Discussion

The essential oil components cineole, carvacrol, and thymol elicited distinct, concentration-dependent responses in chickpea plants. At 1000 ppm, carvacrol significantly enhanced chickpea growth, notably improving fresh and dry biomass. Comparable beneficial effects of carvacrol treatments on plant growth were previously reported in perennial ryegrass (*Lolium perenne* L.), highlighting its protective antioxidative and antimicrobial properties under stress conditions [26]. Conversely, thymol exhibited clear phytotoxicity, significantly reducing plant height, fresh weight, and dry biomass. However, such reductions in height could potentially benefit agricultural systems prone to lodging by enhancing yield stability, a strategy effectively employed during the Green Revolution using growth regulators such as paclobutrazol [62,63]. Interestingly, thymol at 1000 ppm significantly increased chlorophyll content, possibly indicative of a stress-induced protective or hormetic response, a phenomenon documented under moderate stress conditions in other plant species [64]. In contrast, cineole notably decreased chlorophyll content at 1000 ppm, suggesting adverse impacts on the efficiency of photosynthetic machinery.

Variations observed in chickpea mineral concentrations following treatments align with existing research highlighting their influence on nutrient uptake processes. Specifically, thymol at 1000 ppm significantly reduced concentrations of P, K, Mn, Fe, Cu, and Zn. These effects likely arise from thymol’s antimicrobial properties, which disrupt beneficial soil microbial communities essential for nutrient cycling, thus reducing nutrient availability and subsequent uptake by chickpea plants [65]. Additionally, thymol may directly affect root membrane integrity and nutrient transport mechanisms, further limiting mineral absorption [66]. Similar phytotoxic effects were reported by Kordali et al. (2008) in weed species such as *Amaranthus retroflexus*, *Chenopodium album*, and *Rumex crispus* at comparable concentrations [67]. Conversely, Ca concentrations significantly increased with cineole and carvacrol, potentially enhancing plant structural integrity and signaling pathways, consistent with calcium’s established role as a signaling messenger in stress responses [68]. At higher carvacrol concentrations, substantial reductions in P and Ca may result from oxidative stress-induced membrane disruption, impairing nutrient absorption, as previously observed in weed species like *Echinochloa crus-galli* and *Amaranthus retroflexus* [69].

Antioxidant activity in chickpea plants, measured by total phenolic content (TPC) and total flavonoid content (TFC), was positively influenced by cineole and carvacrol. Cineole significantly elevated TPC at 1000 ppm, consistent with findings by Kaur et al. (2020) in spearmint (*Mentha spicata* L.), where cineole-rich essential oils similarly increased phenolic content [70]. Carvacrol also enhanced TPC at the same concentration, aligning with the findings of Saghrouchni et al. (2023), suggesting activation of phenylpropanoid pathways associated with plant stress defense [26]. Increased TPC enhances antioxidant capacities, effectively neutralizing reactive oxygen species (ROS) generated under environmental stress conditions [70,71,72]. In contrast, thymol significantly reduced TPC and TFC at 1000 ppm, echoing observations from Gholami et al. (2021) in *Dracocephalum moldavica*, where high thymol concentrations impaired secondary metabolite biosynthesis [73]. These results indicate a phytotoxic threshold, beyond which secondary metabolism is suppressed, as previously reported by Kleinwächter and Selmar (2015) in medicinal plants including sage (*Salvia officinalis* L.) and thyme (*Thymus vulgaris* L.) [74]. Similar stress-response patterns were documented in chickpeas, where moderate stress stimulates phenolic production, while severe stress negatively impacts metabolism and overall plant vitality [75].

The observed significant decrease in total soluble protein content following treatments, particularly cineole and carvacrol at 1000 ppm, likely resulted from oxidative stress-induced protein damage, causing fragmentation and increased proteolytic activity [76,77]. Such oxidative conditions disrupt enzymatic functions and photosynthetic efficiency [78]. Notably, carvacrol at lower concentrations maintained protein levels close to controls, indicative of mild stress conditions that possibly elicited a beneficial hormetic response, stabilizing protein synthesis and cellular functions, as observed in chickpeas under moderate stress [79,80]. Given chickpea seeds’ inherently high protein content, maintaining protein integrity is critical for nutritional quality and yield [80,81].

Vitamin C content markedly increased under thymol treatment at 1000 ppm, reflecting enhanced antioxidant defenses crucial for mitigating oxidative stress by stabilizing cellular redox status [82,83]. Conversely, the substantial reduction observed with carvacrol at lower concentrations suggests oxidative stress surpassing antioxidant capacities, leading to decreased biosynthesis or enhanced degradation of ascorbate [84]. Given vitamin C’s essential role in chickpea stress tolerance, iron absorption, and nutritional quality [3], the pronounced thymol-induced increase aligns with previous findings in basil (*Ocimum basilicum* L.) and peppermint (*Mentha piperita* L.), where thymol similarly increased antioxidant enzyme activities and vitamin C content under stress conditions [85]. These insights highlight the potential agricultural relevance of optimized thymol application to enhance chickpea nutritional quality and stress resilience.

Thymol and carvacrol, two key components in essential oils, are chemical isomers sharing the same molecular backbone, p-cymene, but differing in the position of the hydroxyl group (OH) [86]. This subtle structural difference significantly influences their distinct biological activities [87]. The positioning of the hydroxyl group affects interactions with biological targets, such as membrane lipids and enzymes, modulating their antimicrobial, antioxidant, and phytotoxic effects [86]. Positional isomerism can notably alter activity profiles, as hydroxyl group placement influences molecular polarity, hydrophobicity, and receptor binding affinity [88]. This structural variation likely explains the distinct physiological responses observed in this study, where thymol and carvacrol exhibited markedly different effects on plant growth and stress-related biochemical markers. These observations, supported by previous structure-activity studies, underscore the importance of molecular structure in determining the biological efficacy of essential oil components, particularly for agricultural applications aimed at optimizing plant health and productivity [87].

Molecular docking served as a pivotal tool to elucidate interactions between the ligands thymol, carvacrol, and cineole with essential protein targets, influencing critical physiological pathways in chickpea plants. Protochlorophyllide Reductase (PDB: 6L1H) participates in the final stages of chlorophyll biosynthesis, significantly impacting photosynthetic capacity and overall plant productivity [89]. Chalcone Synthase (PDB: 1I89) initiates flavonoid biosynthesis, producing phenolic compounds with substantial antioxidant and protective roles [90]. Phenylalanine Ammonia-Lyase (PDB: 6AT7) catalyzes a key step in phenylpropanoid metabolism, influencing numerous defensive phenolic compounds [91]. Ascorbate Peroxidase (PDB: 1IYN) utilizes vitamin C to detoxify reactive oxygen species (ROS), thus protecting cells from oxidative damage [92]. Ferritin (PDB: 3OGH) manages iron storage and regulation, consistent with mineral profiles obtained from XRF analyses [93]. This integrative approach, linking biochemical measurements with computational validation, offers a robust framework to improve plant resilience and quality, clarifying mechanisms underlying the beneficial effects of thymol, carvacrol, and cineole.

In the 2D representation of thymol binding to ascorbate peroxidase (Figure 4), a predominance of hydrophobic and aromatic interactions accounts for the strongly negative docking score (−6.7 kcal/mol). Thymol’s phenolic ring is surrounded by multiple hydrophobic residues (Leu159, Leu258, Ala162, His163), establishing stabilizing alkyl and π-alkyl interactions within the binding pocket [94]. Additionally, Trp33 engages in π-π stacked and π-alkyl interactions, further reinforcing thymol’s binding stability. Polar and aromatic residues such as Pro131, Phe146, Leu142, Tyr251, and Ser160 are positioned at van der Waals distances, subtly yet significantly contributing to the overall stabilization. Although classical hydrogen bonds are not observed, thymol’s hydroxyl group could participate indirectly in hydrogen bonding through structured water molecules or transient dipoles. This combination of hydrophobic and aromatic interactions, supplemented by potential minor polar contributions, imparts substantial thermodynamic stability, explaining the highly favorable docking score. Moreover, this interaction profile is consistent with the enzymatic function of ascorbate peroxidase, whose active site typically features a hydrophobic and aromatic cavity conducive to binding small phenolic compounds. Consequently, the spatial arrangement of these residues around thymol, along with the ligand’s favorable geometry, underpins the predicted high affinity, emphasizing the importance of this protein region in selective ligand recognition.

In the 3D representation of thymol binding to ascorbate peroxidase (Figure 4), the active-site surface is colored according to the solvent-accessible surface (SAS) and hydrogen-bond donor/acceptor potential [95]. Predominantly green regions within the binding pocket indicate extensive hydrophobic or slightly apolar interactions surrounding thymol. In contrast, localized blue or mauve patches highlight polar zones capable of hydrogen bond formation. This surface distribution reinforces earlier observations that aromatic and alkyl interactions (π-π stacking, π-alkyl, and van der Waals) primarily stabilize the complex. Nevertheless, the limited polar surfaces suggest that indirect hydrogen bonding via structured water molecules or weak dipoles may occur with thymol’s hydroxyl group. The dominance of green hydrophobic surfaces complements and confirms the molecular interaction analysis, underscoring the central role of hydrophobic and aromatic interactions in anchoring thymol, while also indicating potential synergy with minor polar interactions.

In Figure 5, the carvacrol–ascorbate peroxidase (1IYN) complex displays interactions similar to those observed with thymol, resulting in a comparable docking score (−6.5 kcal/mol). Most interacting residues, notably hydrophobic and aromatic residues, remain essentially identical, indicating a closely related binding mode within the ascorbate peroxidase catalytic pocket. Slight variations in distances between specific functional groups, typically on the order of tenths of an angstrom, can be attributed to the ortho substitution (isopropyl group) on the aromatic ring. This substitution prompts minimal but adequate spatial adjustments, slightly altering the local ligand orientation. Such subtle variations in side-chain positioning and molecular geometry thus explain the minor differences in docking scores relative to thymol.

In the combined 2D and 3D depiction (Figure 6) of cineole interacting with protochlorophyllide reductase (PDB: 6L1H), analysis reveals ten alkyl-type contacts involving residues Ala A308, Lys A62, Met A59, Val A210, Leu A214, and Pro A307 at distances ranging from approximately 3.86 to 5.27 Å, indicative of significant hydrophobic interactions [96]. These interactions, primarily concentrated around the ether ring and apolar side chains of protein residues, contribute to the relatively negative docking score (−6.1 kcal/mol), signifying notable binding affinity. In the 3D view, the solvent-accessible surface (SAS) map predominantly exhibits blue coloration, suggesting a largely polar environment with regions potentially receptive to water molecules or dipolar interactions. Despite this overall polarity, discrete green hydrophobic pockets accommodate cineole, allowing for effective alkyl interactions that stabilize the ligand. Although direct hydrogen bonds (H-bonds) are rare or absent, indirect bonding via structured water molecules could further enhance ligand binding in certain conformations. Thus, the balance between polar and hydrophobic areas within the binding pocket, combined with cineole’s apolar geometry, results in a stabilizing interaction: robust alkyl contacts firmly anchor the ligand, while the polar pockets may facilitate selective solvation and recognition. Collectively, these factors elucidate the docking score (−6.1 kcal/mol) and emphasize the chemical and spatial complementarity between cineole and protochlorophyllide reductase.

Molecular dynamics is a critical step to further investigate and validate the results obtained from molecular docking and prior biological assays. In this study, we specifically selected the thymol–ascorbate peroxidase (1IYN) complex for a 100 ns molecular dynamics simulation based on a favorable docking score of −6.7 kcal/mol, indicating a strong affinity of thymol for the enzyme’s active site. However, a favorable docking score alone does not guarantee the long-term stability of the complex, emphasizing the necessity of molecular dynamics simulations to confirm the persistence of the identified interactions. This selection is further justified by the prior validation of the docking procedure for the 1IYN protein, which yielded an exceptionally low RMSD value of 0.171 Å, thereby reinforcing the reliability of the results.

**Figure 7:**
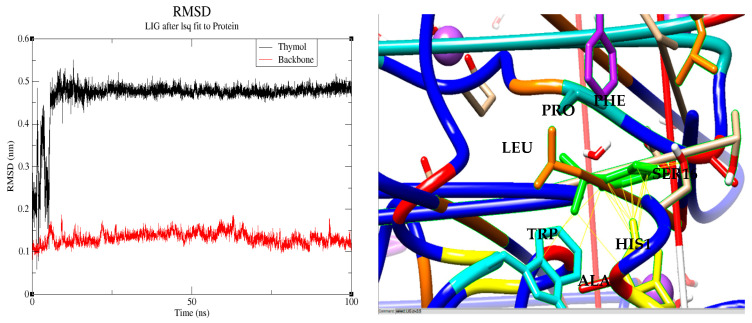
Root mean square deviation (RMSD) analysis of the thymol–1IYN complex over 100 ns (**left**) and detailed molecular interactions between thymol and key active site residues at 90 ns (**right**).

Through molecular dynamics, we precisely monitored the evolution of interactions between thymol and key residues within the enzyme’s active site, focusing on hydrophobic and aromatic contacts (Leu159, Ala162, His163, Trp33) previously highlighted by docking analyses. Over the 100 ns simulation, detailed analyses confirmed the sustained stability of the complex, thereby supporting initial theoretical predictions and experimental data. Specifically, root mean square deviation (RMSD) analysis showed that thymol rapidly stabilized after approximately 10 ns, maintaining minimal fluctuations with an average RMSD value of about 5 Å (0.5 nm) until the end of the simulation (Figure 7). Simultaneously, the protein backbone exhibited remarkable conformational stability, reflected by an RMSD consistently near 1 Å (0.1 nm) throughout the simulation, underscoring the structural robustness of the thymol–ascorbate peroxidase complex [97].

At 90 ns, thymol forms strong and direct interactions (distance < 3.5 Å) with several critical residues in the enzyme’s active site, including His163, Ala162, Trp33, and Leu159, thereby validating the initial docking predictions. Notably, His163 is recognized as the proximal histidine responsible for coordinating iron in numerous peroxidases, thus playing a crucial catalytic role [98]. Additionally, thymol is closely surrounded by other significant residues such as Pro131, Ser160, and Phe146, all within distances below 3.5 Å, in perfect agreement with docking results.

The convergence of these proximal, stable interactions and the retention of thymol’s predicted binding orientation affirm the robustness and accuracy of its anchoring within the 1IYN active site. Ultimately, these observations highlight the importance of differentiating key catalytic residues, such as His163, from those primarily involved in structural support or substrate stabilization (Ala162, Trp33, Leu159). Consequently, the remarkable consistency between docking predictions and molecular dynamics outcomes substantially strengthens conclusions about the affinity and stability of the thymol–1IYN complex. The exceptional stability observed underscores its significant biological potential, particularly regarding the management of oxidative stress in plants.

**Figure 8:**
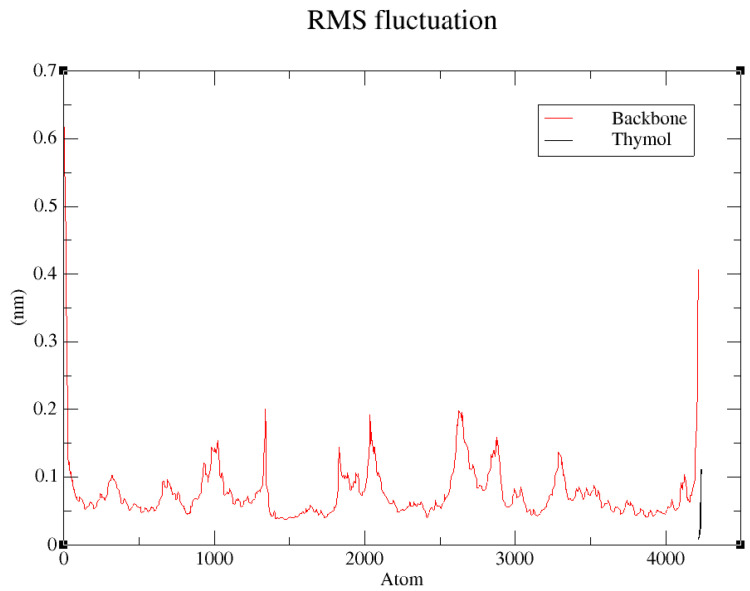
RMS mean square fluctuation (RMSF) analysis of backbone atoms in the thymol–1IYN complex during the 100 ns molecular dynamics simulation.

The root mean square fluctuation (RMSF) profile for the backbone atoms of the thymol–1IYN complex provides a precise measurement of local flexibility across each region of the protein throughout the 100 ns molecular dynamics simulation (Figure 8). Overall, most residues exhibit moderate fluctuations, typically remaining below 0.1 nm, confirming the remarkable global stability of the protein backbone. Nonetheless, several isolated peaks are evident, reaching approximately 0.15–0.2 nm [99]. These peaks primarily correspond to inherently flexible regions, such as loops located on the protein surface. Significantly, the active-site residues interacting directly with thymol show notably low RMSF values, reflecting substantial structural rigidity in these crucial areas. Such findings strongly imply that thymol binding stabilizes the active site by effectively constraining its flexibility, further supporting the reliability of previous molecular docking and RMSD analyses. Conversely, more pronounced fluctuations are observed at the protein’s terminal regions, particularly near residue indices around atom 4000. This behavior aligns with expectations, as terminal segments generally exhibit lower structural organization and greater exposure to solvent dynamics.

Collectively, the RMSF analysis clearly confirms the high stability of the thymol–1IYN complex, particularly within the critical active-site region. These results robustly validate prior observations and reinforce the scientific credibility of the interactions identified between thymol and the target protein.

Understanding the physiological responses of chickpea plants to various treatments is essential for enhancing agricultural productivity. Key biochemical parameters, such as phenolic content, mineral nutrient levels, and antioxidant activity, play pivotal roles in photosynthesis, secondary metabolism, and stress tolerance [19]. For instance, an increase in phenolic compounds induced by essential oil treatments can significantly mitigate oxidative stress, thereby enhancing yield stability [73]. Similarly, optimal phosphorus, potassium, and calcium concentrations are essential for metabolic efficiency and structural integrity, directly influencing productivity [100]. Studying these biochemical pathways provides valuable insights for developing resilient and high-yielding chickpea cultivation strategies.

## 5. Conclusions

Foliar applications of cineole, carvacrol, and thymol significantly influenced chickpea growth, nutrient uptake, and antioxidant metabolism in a concentration-dependent manner. Carvacrol at 1000 ppm notably enhanced fresh weight, biomass accumulation, and antioxidant capacity. Conversely, thymol exhibited phytotoxic effects at higher concentrations (1000 ppm), although it positively influenced chlorophyll content and markedly elevated vitamin C levels, indicating an adaptive antioxidant response. Cineole treatment increased phenolic and flavonoid contents, thus enhancing overall antioxidant activity; however, higher doses negatively impacted chlorophyll biosynthesis and protein synthesis.

Molecular docking analyses demonstrated strong binding interactions of thymol and carvacrol with critical antioxidant enzymes, particularly phenylalanine ammonia-lyase and ascorbate peroxidase. Moreover, stable thymol–enzyme complexes identified by molecular docking were corroborated by 100 ns molecular dynamics simulations, suggesting potential roles in mitigating oxidative stress. Although the current investigation primarily addressed chickpea physiological and biochemical responses, further field studies are essential to elucidate broader implications for crop yield and agronomic performance. Future research should also aim to clarify the molecular mechanisms underlying monoterpene actions across diverse plant species and environmental contexts. Overall, our findings position carvacrol and thymol as promising natural growth regulators with substantial potential for sustainable crop management. These monoterpenes hold promise as eco-friendly biostimulants capable of enhancing plant productivity and resilience under abiotic stress conditions.

## Figures and Tables

**Table 1:** Concentrations of mineral elements (mg kg^−1^) in chickpea plants subjected to foliar application of selected monoterpenes at two concentrations. Values are presented as mean ± SE (n = 6). Within each row, values followed by different superscript letters differ significantly (*p* < 0.05) according to Tukey’s post hoc test.

Mineral Elements	Control	Cineole		Carvacrol		Thymol	
		500 ppm	1000 ppm	500 ppm	1000 ppm	500 ppm	1000 ppm
**P**	8716 ± 68 ^ab^	8313 ± 56 ^c^	8870 ± 61 ^a^	7471 ± 49 ^d^	6797 ± 28 ^e^	8553 ± 27 ^b^	7000 ± 17 ^e^
**S**	3920 ± 21 ^a^	3376 ± 43 ^b^	2704 ± 33 ^e^	2854 ± 21 ^d^	3243 ± 16 ^c^	3196 ± 25 ^c^	2967 ± 27 ^d^
**Cl**	1769 ± 20 ^d^	2093 ± 12 ^b^	2352 ± 17 ^a^	2153 ± 17 ^b^	1923 ± 11 ^c^	1872 ± 16 ^c^	1411 ± 13 ^e^
**K**	27,445 ± 130 ^a^	26,356 ± 163 ^b^	23,495 ± 93 ^d^	24,634 ± 60 ^c^	23,051 ± 91 ^d^	26,252 ± 81 ^b^	21,531 ± 113 ^e^
**Ca**	7649 ± 39 ^c^	8303 ± 45 ^b^	9984 ± 39 ^a^	10,096 ± 39 ^a^	6789 ± 19 ^d^	9944 ± 36 ^a^	6758 ± 27 ^d^
**Mn**	45.1 ± 0.5 ^b^	36.7 ± 0.7 ^c^	39.7 ± 0.8 ^c^	48.4 ± 0.3 ^a^	29.9 ± 0.6 ^d^	45.0 ± 1.1 ^b^	22.9 ± 0.7 ^e^
**Fe**	60.7 ± 2.3 ^a^	38.4 ± 0.9 ^cd^	44.0 ± 1.3 ^bc^	49.9 ± 1.5 ^b^	36.5 ± 1.7 ^d^	48.7 ± 1.5 ^b^	29.6 ± 0.2 ^e^
**Cu**	3.6 ± 0.4 ^a^	3.2 ± 0.2 ^ab^	2.4 ± 0.2 ^ab^	3.2 ± 0.3 ^ab^	3.0 ± 0.2 ^ab^	3.5 ± 0.2 ^ab^	2.3 ± 0.1 ^b^
**Zn**	50.7 ± 0.4 ^c^	48.2 ± 0.5 ^c^	53.8 ± 0.3 ^b^	57.8 ± 1.0 ^a^	41.4 ± 1.0 ^d^	58.9 ± 0.6 ^a^	29.5 ± 0.6 ^e^

**Table 2:** Comparative docking scores (kcal/mol) of thymol, carvacrol, and cineole against five key enzyme targets.

Molecules	Docking Score (kcal/mol)				
	6L1H	1I89	6AT7	1IYN	3OGH
Thymol	−6.4	−6.1	−6.7	−6.7	−5.3
Carvacrol	−6.2	−5.1	−6.5	−6.5	−5.3
Cineole	−6.1	−5.2	−5.4	−5.7	−4.7

## Data Availability

The raw data are available from the corresponding author.
